# Genetic Factors of Autoimmune Thyroid Diseases in Japanese

**DOI:** 10.1155/2012/236981

**Published:** 2011-12-27

**Authors:** Yoshiyuki Ban

**Affiliations:** Division of Diabetes, Metabolism, and Endocrinology, Department of Medicine, Showa University School of Medicine, 1-5-8 Hatanodai, Shinagawa-ku, Tokyo 142-8666, Japan

## Abstract

Autoimmune thyroid diseases (AITDs), including Graves' disease (GD) and Hashimoto's thyroiditis (HT), are caused by immune response to self-thyroid antigens and affect approximately 2–5% of the general population. Genetic susceptibility in combination with external factors, such as smoking, viral/bacterial infection, and chemicals, is believed to initiate the autoimmune response against thyroid antigens. Abundant epidemiological data, including family and twin studies, point to a strong genetic influence on the development of AITDs. Various techniques have been employed to identify genes contributing to the etiology of AITDs, including candidate gene analysis and whole genome screening. These studies have enabled the identification of several loci (genetic regions) that are linked to AITDs, and, in some of these loci, putative AITD susceptibility genes have been identified. Some of these genes/loci are unique to GD and HT and some are common to both diseases, indicating that there is a shared genetic susceptibility to GD and HT. Known AITD-susceptibility genes are classified into three groups: HLA genes, non-HLA immune-regulatory genes (e.g., CTLA-4, PTPN22, and CD40), and thyroid-specific genes (e.g., TSHR and Tg). In this paper, we will summarize the latest findings on AITD susceptibility genes in Japanese.

## 1. Introduction

Autoimmune thyroid diseases (AITDs) are common autoimmune endocrine diseases [1], and according to one study, AITD are the commonest autoimmune diseases in the USA [2]. Even though the hallmark of AITD is infiltration of the thyroid with thyroid reactive lymphocytes, the end result is two clinically opposing syndromes: Hashimoto's thyroiditis (HT) manifesting by hypothyroidism and Graves' disease (GD) manifesting by hyperthyroidism. In HT, the lymphocytic infiltration of the thyroid gland leads to apoptosis of thyroid cells and hypothyroidism [3]. In contrast, in GD, the lymphocytic infiltration of the thyroid leads to activation of TSH-receptor- (TSHR) reactive B cells that secrete TSHR-stimulating antibodies causing hyperthyroidism [4]. GD and HT are complex diseases, and their etiology involves both genetic and environmental influences [1]. Up until 15 years ago, the only known gene for AITD was HLA-DR3 haplotype (DRB1*03-DQB1*02-DQA1*0501) in Caucasians. However, with the advent of new genomic tools and the completion of the human genome and the HapMap projects, new non-HLA genes have been identified and their functional effects on disease aetiology started to be dissected as well. This paper will summarize the recent advances in our understanding of the genetic contributions to the etiology of AITD in Japanese population. Since most of the studies were performed in relatively small size samples recruited from Japanese population, the results have some limitations. 

## 2. A Brief Overview of AITD Genes Identified in Caucasians

In Caucasians, the first locus shown to be associated with AITDs was the HLA-DRB1 locus (reviewed in [5]). HLA-DR3 (DRB1*03) haplotype has been consistently shown to be associated with GD, with an odds ratio (OR) of 2.0–3.0 [6–8]. The literature regarding HT is less consistent with reports of associations with DR3 and DR4 in Caucasians, as well as a negative association with DR 1 and 8, suggesting a protective role [9]. Recently, Zeitlin et al. [10] investigated DRB1-DQB1-DQA1 in the largest UK Caucasian HT case control cohort to date comprising 640 HT patients and 621 controls. A strong association between HT and DR4 haplotype (DRB1*04-DQB1*03-DQA1*03) was detected, and protective effects were detected for DR13 haplotype (DRB1*13-DQB1*06-DQA1*01) and DR7 [10]. It was recently shown that arginine at position 74 of the DR*β*1 chain (DR*β*1-Arg74) is important for the development of GD in a significant proportion of patients [11, 12]. A study from England provided evidence of a primary association of HLA-C, and, to a lesser extent HLA-B, with GD. Other genes have also been shown to influence the expression of GD in Caucasians [13]. These include the genes for cytotoxic T-lymphocyte-associated protein 4 (CTLA-4) [14, 15], CD40 [16], protein tyrosine phosphatase-22 (PTPN22) [17], thyroglobulin (Tg) [18, 19], and TSH-receptor (TSHR) genes [20].

## 3. HLA Genes in Japanese

Located on chromosome 6p21 is the major histocompatibility complex region that encodes for HLA glycoproteins. The HLA region is a highly polymorphic region that contains many immune response genes and has been found to be associated with various autoimmune disorders. The HLA molecule binds a peptide antigen (autoantigen in the cause of autoimmunity). It presents the antigen for recognition by the T-cell and as such the T-cell then determines if the antigen is self (and no immune response is mounted) or nonself and an immune response is mounted [21]. 

The HLA associations are with different alleles in Japanese. In previous studies, HLA-B35 is associated with GD and HLA-DRw53 with HT in the Japanese population (reviewed in [22]). HLA-Bw46 is associated with GD and HLA-DR9 with HT in the Chinese population (reviewed in [22]). The European GD-associated HLA haplotype (HLA-B*08-DRB1*03-DQA1*0501-DQB1*02) is virtually absent in Japanese [23]. Dong et al. previously reported that HLA-A*02 and DPB1*0501 are associated with Japanese GD [24]. Recently, they also demonstrated that HLA-A*02 and DPB1*0202 showed association with thyroid-stimulating hormone-binding inhibitory immunoglobulins- (TBII) negative GD, indicating that TBII-negative GD may be genetically distinct from TBII-positive GD [25]. In addition, Wan et al. reported that HLA-A*02 and DPB4*0101 are associated with Japanese HT [26].

## 4. Non-HLA Immune-Regulatory Genes in Japanese

### 4.1. The CTLA-4 Gene

The cytotoxic T-lymphocyte-associated protein 4, CTLA-4, gene is located on chromosome 2q. It is a highly polymorphic gene that was first discovered to be associated with risk for AITD by the candidate gene approach. Under normal circumstances, the CTLA-4 protein acts to suppress T-cell activation during normal immune response in order to prevent T-cell overactivity [27]. CD4+CD25-T-cells only express CTLA-4 on their surface after the T-cell receptor is activated, and its engagement with its ligand suppresses the ongoing immune response. Decreased or absent CTLA-4 activity permits uninhibited T-cell activity and a prolonged, unregulated immune response [28], making CTLA-4 an attractive candidate gene for autoimmunity. Indeed, the CTLA-4 gene has been found to be associated with many other autoimmune diseases. 

A microsatellite in 3′UTR of CTLA-4 has been linked to AITD in Caucasians (reviewed in [27]); the longer the AT repeat at this site is, the less inhibitory activity CTLA-4 has. It has also been associated with AITD in Japanese [29]. Other variants of CTLA-4 gene have been linked to AITD in Caucasians (reviewed in [27]); a G allele substitution at an A/G single nucleotide polymorphism (SNP) at position 49 was also found to be associated with AITD in Japanese [30]. Recently, an A/G SNP downstream from the 3′UTR, designated CT60, was found to be associated with GD in Caucasians and has been suggested as the causative variant, albeit this has not been conclusively demonstrated [15]. It was also found to be associated with AITD in Japanese [31, 32]. Interestingly, another SNP (rs231779) is more likely the susceptibility variant for GD in Chinese Han population, suggesting that the susceptibility variants of the CTLA-4 gene varied between the different geographic populations with GD [33]. Additionally, most recent stratification analyses suggested a possible synergistic interaction of CTLA-4 CT60 with HLA-A*02 and -DPB1*0501 in the susceptibility to TBII-positive GD [34].

### 4.2. The CD40 Gene

The CD40 molecule, located on chromosome 20q, is crucial to both innate adaptive immune responses. It is present on the surface of antigen presenting cells (APCs) including B cells. The T-cell-APC interaction results in activation of CD40 as a costimulatory molecule. CD40 also plays a critical role in activating B lymphocytes allowing them to terminally differentiate and secrete antibodies (reviewed in [35]). It is no surprise that the CD40 gene has been linked to many autoimmune disorders. Whole genome linkage scanning has identified strong linkage of CD40 to GD. The causative variant predisposing to GD is a C/T polymorphism in the Kozak sequence (dbSNP accession number rs1883832), a nucleotide sequence that is essential for the initiation of translation of the CD40 molecule. Specifically, the CC genotype has been identified in Caucasians to be associated with GD [16]. Indeed, functional studies demonstrated that the C-allele of this SNP increased CD40 mRNA translation by *∼*20–30% when compared with the protective T allele [27]. We and others have also confirmed an association between the rs1883832 and GD in Japanese [36, 37].

### 4.3. The PTPN22 Gene

The protein tyrosine phosphatase-22 (PTPN22) gene encodes for the lymphoid tyrosine phosphatase (LYP), a molecule that, similar to CTLA-4, functions to inhibit T-cell activation [38]. A nonsynonymous SNP in the PTPN22 gene, R620 W (dbSNP accession number rs2476601), was found to be associated with GD, as well as other autoimmune diseases. This substitution results in a functional change in the LYP protein resulting in activation of T-cell, but the mechanism is unclear [35]. Indeed, this association seems specific for Caucasians and was not found in the Japanese GD population [39]. 

Recently, the rs2488457 SNP in the promoter region was reported to be associated with acute onset T1D in a Japanese population [40]. However, there was no association of the rs2488457 SNP with GD [41]. Furthermore, the rs3789604 SNP of the PTPN22 gene was found to be associated with RA, independently of rs2476601 [42]. The rs3789604 SNP lies 1496 bases downstream of PTPN22 at the 50 end of the round spermatid basic protein 1 gene (RSBN1), where it encodes either a silent mutation or putative transcription factor-binding sites (TRBS), depending on the transcript. Recently, the AA-genotype and A-allele frequencies of the rs3789604 were significantly higher in GD patients than in control subjects [43], suggesting that the rs3789604 or a gene with linkage disequilibrium may be relevant to susceptibility to GD in Japanese populations. Therefore, we further analyzed five other SNPs including rs12760457, rs2797415, rs1310182, rs2476599, and rs3789604, to clarify whether a susceptibility locus for AITD exists at another location within the PTPN22 gene. Our results showed no association with disease of any of the individual SNPs [44].

Because of the strong LD between five variants, haplotype analysis was undertaken using the computer program SNPAlyze version 7.0. Five haplotypes were identified, three of which (haplotypes 1, 2, and 4) were correlated with haplotypes 1, 4, and 5 identified in the report by Carlton et al. (Table 1) [42]. Four haplotypes (haplotypes 1–4) were relatively common, and 1 haplotype was rare. Distribution of the haplotype is significantly different between AITD and control by permutation procedure (*P* = 0.0036) [44]. A novel protective effect of a haplotype containing five SNPs was observed (*P* < 0.0001 for AITD, *P* < 0.0001 for GD, and *P* < 0.0001 for HT, resp.) (Table 1) [44].

### 4.4. The Zinc-Finger Gene in the AITD Susceptibility Region (ZFAT) Gene

Shirasawa et al. [45] identified a novel zinc-finger gene, designated ZFAT, as one of the AITD susceptibility genes in 8q23-q24 through an initial association analysis using the probands in their previous linkage analysis [46]. The distance between thyroglobulin and ZFAT genes is about 1.8 M bp. A subsequent association analysis of the samples from a total of 515 affected individuals and 526 controls in Japanese [45]. The T allele of the SNP located in the intron 9 of ZFAT (Ex9b-SNP10) is associated with increased risk for AITDs (dominant model: OR = 1.7, *P* = 9.1 × 10^−5^) [45]. The Ex9b-SNP10 is located in the 3′-UTR of truncated-ZFAT (TR-ZFAT) and the promoter region of the SAS-ZFAT [45]. The human ZFAT gene encodes a 1,243-amino acid residue protein containing one AT-hook and 18 C2H2 zinc-finger domains. ZFAT is also highly conserved among species from fish to human [44]. The ZFAT protein is expressed in the B and T lymphocytes in mice, and ZFAT regulates the genes involved in immune responses [47]. Furthermore, ZFAT is an anti-apoptotic molecule that is critical for cell survival in human leukemic MOLT-4 cells [48]. 

### 4.5. The FCRL Genes

Fc receptor-like 3 (FCRL3) is one of five FCRL genes that are preferentially expressed on B lymphocytes and have a highly structural homology with Fc receptors [49]. The 1p21-23 cytoband, in which the FCRL family resides, has been identified as a candidate locus for multiple autoimmune disorders in both human and murine models [50]. Kochi et al. [51] identified a strong association of SNPs in this region with GD susceptibility in Japanese and concluded that the origin of the association was a regulatory SNP in the promoter region of FCRL3. This susceptibility gene of GD was first identified from Japanese population. This SNP (−169C/T) (dbSNP accession number rs7522061) alters the binding affinity of NF*κ*B and regulates gene expression, and high FCRL3 expression on B lymphocytes is observed in individuals with the disease-susceptible genotype. The SNP rs7522061 in the FCRL3 gene was also reported to be associated with AITD in Caucasians [52] and two other autoimmune diseases, rheumatoid arthritis (RA), and systemic lupus erythematosus (SLE) [51]. More recently, SNP rs3761959, which tags rs7522061 and rs7528684 (previously associated with RA and GD), was associated with GD in the extended cohort, confirming the original result. In total, three of the seven FCRL3 SNPs showed some evidence for association (*P* < 0.05), with SNP rs11264798 showing the strongest association of the tag SNPs (*P* = 4.0 × 10^−3^) [53]. SNP rs6667109 in FCRL5, which tagged SNPs rs6427384, rs2012199, and rs6679793, all found to be weakly associated in the original study, showed little evidence of association in the extended cohort [53].

### 4.6. Other Immune-Regulatory Genes

Other immune-regulatory genes tested for association with AITD in Japanese include Interleukin-23 receptor (IL-23R) [54], Interferon-induced helicase (IFIH1) [55], FOXP3 [56], and interleukin-2 receptor alpha (IL-2RA) genes [44]. 

Interleukin-23 (IL-23) is a recently discovered heterodimeric cytokine. IL-23 acts primarily on CD4+ T cells that have already been exposed to antigens, sustaining long-term cellular immunity by promoting survival and the effector cytokine production of T helper 1 (Th1) memory cells [57]. Several SNPs in the IL-23R gene have recently been shown to be associated with autoimmune and inflammatory conditions, including Crohn's disease [58], rheumatoid arthritis (RA) [59], and psoriasis [60]. More recently, an association was reported between these SNPs and GD and Graves' ophthalmopathy (GO) in North American Caucasians [61]. SNP rs11209026 is specific for Crohn's disease [58], while rs10889677 and rs2201841 have been shown to confer risk for both Crohn's disease [58] and rheumatoid arthritis (RA) [59]. Intriguingly, these latter two SNPs are the same SNPs Huber et al. [61] found to be associated with GO; however, rs7530511 was associated with GD, but not specifically with GO [61]. In contrast, the rs11209026 SNP did not show an association with GD or GO in their cohort [61], but this SNP has not been proven to be the causative SNP in Crohn's disease [58]. Thus, different variants in the IL-23R gene may predispose individuals to different autoimmune conditions. We did not find an association between the IL-23R gene and AITDs in the Japanese population, perhaps due to ethnic differences, environmental factors, or a very small effect that cannot be detected in our dataset [54].

Interferon-induced helicase 1 (IFIH1) is thought to have a role in protecting the host from viral infection by sensing viral nucleic acids in the cytoplasm and triggering a cellular antiviral and apoptotic response [62, 63]. Because Coxsackie and other enteroviral infections are epidemiologically linked to type 1 diabetes (T1D) incidence [64], it was suggested that IFIH1 polymorphism acts as a molecular link between the specific viral trigger and the autoimmune response in T1D [65]. Recently, using preliminary results from large-scale association analyses of nonsynonymous SNPs, a novel locus for T1D was identified; IFIH1, which is also known as the melanoma a differentiation-associated 5 (MDA-5), or Helicard, gene [65]. Using logistic regression analysis, the most strongly associated marker in IFIH1 was identified as SNP rs1990760, which encodes an alanine to threonine amino acid change at codon 946, with an odds ratio for association with T1D of 1.16 (5%–95% confidence interval, 1.11–1.22) for the major allele [65]. More recently, the association of IFIH1 alleles with GD in Caucasians was found to be stronger than that with T1D, with an OR for association of 1.47, in contrast to the 1.16 seen for T1D [66]. However, this was not found when IFIH1 was also investigated in a larger independent GD dataset [67]. The IFIH1 gene encodes transcripts that have widespread expression in lymphoid and other tissues, thus suggesting that they could have a role in numerous autoimmune conditions. However, our data did not show significant differences in allele or genotype frequencies for the rs1990760 SNP between AITD patients and control subjects in Japanese [55]. 

Two whole genome scans for linkage in GD have shown evidence for linkage at putative X-chromosome loci, Xq21 [68], and Xp11 [69], and these loci have also been identified in localized linkage scans of the X-chromosome, Xq21 [68] and Xp11 [70], although one of the two genome wide screen increased their numbers and performed an enlarged genome wide screen and no evidence for Xp21 as a region of linkage to GD [71]. In terms of broader relevancy to autoimmunity in general, Xp11 has also been linked to other autoimmune disorders, T1D, multiple sclerosis, and RA, thus suggesting the presence of common susceptibility polymorphism(s) [72–74]. The FOXP3 gene is located at Xp11.23 within this area of autoimmune disease linkage and is therefore an excellent positional candidate gene for autoimmunity at this locus. Indeed, Bassuny et al. [75] reported an association between a functional microsatellite polymorphism, (GT)n, located in the promoter/enhancer region of FOXP3, and T1D in a Japanese population. However, a subsequent study could not confirm the FOXP3 association with T1D in an Italian population [76]. A recent study from the UK tested several FOXP3 polymorphisms for associations with GD and found no robust evidence that those polymorphisms contributes to the susceptibility to GD [77]. We tested the FOXP3 gene locus for associations with AITDs in two cohorts of US Caucasians and Japanese AITD patients [56]. Our study demonstrated a weak association between polymorphisms of the FOXP3 gene and AITD in US Caucasians but not in the Japanese. However, one group from Japanese reported that the −3279A/C SNP of the FOXP3 gene is related to the development and intractability of GD and the −2383CC genotype to the severity of HT [78]. These results, if replicated, may suggest that inherited abnormalities of Treg function may contribute to the etiology of AITD.

The interleukin-2 receptor-*α* (IL-2RA) encodes the *α*-chain of the IL-2 receptor (IL-2R) complex (also known as CD25), which is central to immune regulation as an important modulator of self-tolerance and immunity [79], and the IL-2RA association with type 1 diabetes was originally identified by Vella and coworkers [80]. The IL-2RA gene has also been associated with GD [81], RA [82], and multiple sclerosis [83] in Caucasians, which implies that this locus may have a general effect on predisposition to autoimmunity. Recently, two SNPs in intron 1 of the IL-2RA, rs706778, and rs3118470, were associated with T1D in the Japanese population [84]. However, we did not find an association between the IL-2RA gene and AITDs in the Japanese population [44]. It may be due to the effect size being smaller than that seen in Caucasians or that there dataset may not be powerful enough to detect such an effect.

## 5. Thyroid-Specific Genes in Japanese

### 5.1. The Thyroglobulin Gene

The thyroglobulin (Tg) protein is the major thyroidal protein antigen and is a precursor to thyroid hormones. Tg is also a key antigen in AITD as evidenced by the fact HT is characterized by antithyroglobulin antibodies which are detected in 75% of patients [35]. Whole genome linkage studies identified a locus on chromosome 8q24 that was linked with AITD; this locus contained the Tg gene [71]. Sequencing of the Tg gene identified several nonsynonymous SNPs that were associated with AITD [85]. In our dataset of Japanese AITD patients we found an association between a Tg microsatellite polymorphism and HT [86].

### 5.2. TSH Receptor (TSHR) Gene

The TSHR gene is located on chromosome 14q. It was found to be associated with GD both by the candidate gene approach and by whole genome linkage studies [71]. The TSHR gene was a prime candidate gene for GD since GD is caused by autoantibodies that bind to and stimulate the TSH-receptor. Several TSHR SNPs have been tested for association with GD, including nonsynonymous SNPs in the extracellular TSH-receptor domain and in the intracellular domain of the TSHR; all of these gave conflicting results [87]. However, linkage studies demonstrated significant evidence for linkage of GD with a locus on chromosome 14q harbouring the TSHR gene and many other genes [71]. It was later found that noncoding SNPs in intron 1 of the TSHR confer the association with GD [88]. Recently, association within the TSHR intron 1 in Caucasians was narrowed down to two SNPs (rs179247 and rs12101255) within intron 1 [89]. The disease-associated genotypes of rs179247 (AA) and rs12101255 (TT) showed reduced mRNA expression ratios of flTSHR relative to two alternate TSHR mRNA splice variants [89]. Płoski et al. [90] validated association of TSHR intron 1 SNPs with GD in three independent European cohorts and demonstrated that the etiological variant within the TSHR is likely to be in strong linkage disequilibrium with rs12101255. In Japanese, multiple SNPs in intron 7 of TSHR gene were associated with GD [91].

## 6. Conclusions and Future Direction

Through the genetic studies undertaken to date, we now know that substantial ethnic differences exist in AITD genetic predisposition between populations of Caucasian and Japanese ancestries. Beside the HLA-DRB1 alleles as described, the most evident ethnic difference is seen in the nonsynonymous coding polymorphism of the PTPN22 gene in Caucasian populations. This polymorphism is rarely found in Japanese population [39] and thus specifically contributes to AITD in populations of Caucasian descent. The absence of a disease-risk allele in a population, as in the case of PTPN22, can easily explain the genetic heterogeneity between populations [92]. However, the situation is more complex in cases where the disease-risk allele is shared among different populations and the results of association tests are not. This could occur when (1) a positive association in the primary report represents a false positive due to sampling biases, (2) a negative association observed in a replication study is a false negative due to a lack of statistical power, or (3) true genetic heterogeneity exists (genetic contribution of the gene polymorphisms is zero in a population, or lower than that of the population originally reported) [92]. 

The most recent step in the evolution of genetic studies in common disease sees association analysis performed at a genome-wide level to produce genome-wide association studies [93–95]. These studies, now beginning to emerge in some common diseases, bring together the latest advances in our genetic maps, large-scale automated genotyping technologies, and large national DNA collections [96–98]. Genotyping technology is now being utilized which is able to produce information on over 500000 SNPs in cohorts of between 2000 and 3000 samples. These studies will not only help identify novel loci but should also provide insights into the different technical, analytical, methodological, and biological aspects of genome-wide association analysis. Assuming that these new approaches deliver some novel loci, an important next step will be the validation and replication of loci that will then justify more detailed fine mapping and functional analyses. Recently, one group from China did the first >500 k published SNP screen in a large Chinese population [99]. They identified two new susceptibility loci for atopic dermatitis in the Chinese Han population [99]. Since genetic heterogeneity of disease susceptibility variants between ethnic groups is common in complex diseases, following up this work will be important in the future.

Genetic analyses undertaken in the last decade have revealed a completely new picture of AITD pathogenesis and made us aware of heterogeneity among individuals and populations. Our final goal is to establish new treatments for AITD, based on the pathogenesis and prognosis of individuals, which could lead to the development of tailor-made therapies for AITD. To reach this goal, we should continue to uncover unknown genetic predispositions and clarify differences in roles among ethnicities. Upcoming genome-wide scans for additional populations worldwide and the meta-analysis of these studies may elucidate the complete picture of AITD.

## Figures and Tables

**Table 1:** PTPN22 haplotype structure and frequencies^a^.

	SNP ID					Haplotype comparison^b^						
Haplotype	1	2	3	4	5	AITD	GD	HT	Controls	AITD versus Controls *P* value	GD versus Controls *P* value	HT versus Controls *P* value
1	C	T	T	G	A	0.59	0.59	0.59	0.60	0.69	0.75	0.69
2	C	C	T	G	C	0.20	0.20	0.20	0.17	0.24	0.29	0.28
3	C	C	C	A	A	0.13	0.13	0.13	0.12	0.54	0.66	0.47
4	T	C	C	G	A	0.067	0.066	0.070	0.060	0.63	0.72	0.61
5	C	C	T	G	A	0.013	0.017	0.0061	0.051	<0.0001	<0.0001	0.0004

^a^The program, SNPAlyze ver. 7.0 Standard, was used to estimate common (frequencies > 0.01) haplotypes for the five SNPs genotyped.

^b^Each haplotype was compared with the other haplotypes combined.

SNP: single nucleotide polymorphism; AITD: autoimmune thyroid disease; GD: Graves' disease; HT: Hashimoto's thyroiditis.
